# Insights into Swallowing Disorders: the Dysphagia Course Series

**DOI:** 10.25122/jml-2023-1032

**Published:** 2023-12

**Authors:** Stefana-Andrada Dobran, Alexandra Gherman, Dafin Muresanu

**Affiliations:** 1 RoNeuro Institute for Neurological Research and Diagnostic, Cluj-Napoca, Romania; 2 Sociology Department, Babes-Bolyai University, Cluj-Napoca, Romania; 3 Department of Neuroscaience, Iuliu Hatieganu University of Medicine and Pharmacy, Cluj-Napoca, Romania

## THE SILENT STRUGGLE OF DYSPHAGIA

As one of the most prevalent complications following a stroke, dysphagia manifests as a swallowing disorder that makes it challenging for the affected individuals to eat and drink, posing a significant negative impact on the quality of life. Dysphagia can have many causes and appears with affections of the nervous systems, such as stroke, cerebral palsy, or Parkinson’s disease, profoundly impacting patients’ lives.

Effectively managing dysphagia requires a comprehensive, multidisciplinary approach, uniting the expertise of medical professionals, nutrition specialists, and speech-language pathologists, to ensure a higher quality of life for patients. Consequently, there is a need to enhance knowledge and build management skills in healthcare professionals to ensure better patient outcomes.

## NAVIGATING SWALLOWING DISORDERS: INSIGHTS FROM THE FIRST DYSPHAGIA COURSE SERIES

**Figure 1:**
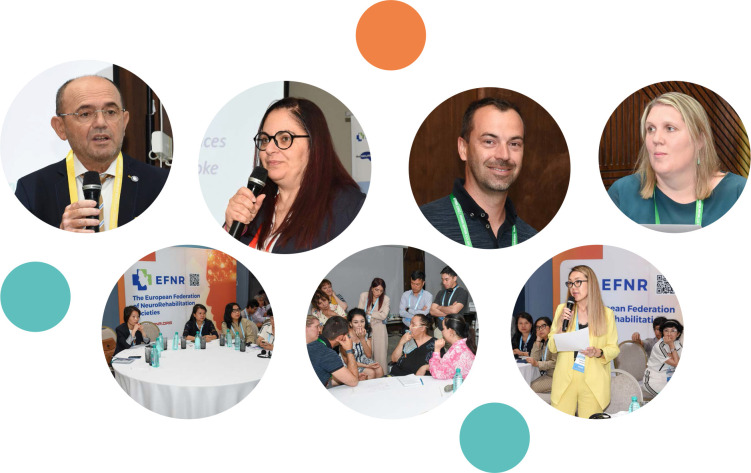
The first Dysphagia Course Series: Profesor Dafin Mureşanu, EFNR President, Francesca Pezzella, Co-Chair Stroke Action Plan for Europe, and the course trainers, Björn Degen and Prof. Stefanie Duchac (first row), along with pictures from the event (second row).

The first Dysphagia Course Series took place in the vibrant Tashkent, in Uzbekistan, on June 22^nd^ and 23^rd^, 2023. The event was organized under the umbrella of the European Federation of Neurorehabilitation Societies (EFNR), represented by its president, Prof. Dr. Dafin F. Muresanu, and the European Stroke Organisation (ESO), represented by Dr. Francesca Pezzella, Co-Chair for the Stroke Action Plan for Europe (SAP-E), showcasing their unwavering commitment to tackling dysphagia head-on (Figure 1). ESO has endorsed the Course Series, signing a memorandum of understanding with EFNR to strengthen further collaboration on education, research, and medical practice in neurorehabilitation. Two exceptional trainers, Prof. Stefanie Duchac, Professor of Speech Therapy at the SRH University of Applied Health Science in Germany, and Mr. Björn Degen, Speech and Language Therapist at the Center for Swallowing Disorders in Vienna, Austria, facilitated the course (Figure 1). Prof. Stefanie Duchac holds extensive clinical experience as a speech therapist specializing in swallowing disorders. Notably, she launched a digital education portal dedicated to dysphagia, as well as a mentorship program in Germany. Mr. Björn Degen’s contributions to the field are supported by his role as a board member of the European Society for Swallowing Disorders and his active participation in scientific events, to enhance awareness and elevate the standard of care for individuals with swallowing disorders.

Participants delved into the profound impact of dysphagia on rehabilitation, exploring its potential complications (e.g., dehydration, malnutrition, and pneumonia), as its management in healthcare settings plays a crucial role in patient well-being. Captivating presentations and interactive demonstrations provided a comprehensive understanding of normal swallowing as the foundation for understanding the pathophysiology, while case examples allowed participants to capture insights into the pathological mechanisms and symptoms of swallowing disorders, with a specific focus on post-stroke dysphagia, a commonly encountered consequence of stroke. The event delved into the identification of swallowing disorders exploring screening tools, as well as their advantages, disadvantages, and clinical utility, with an emphasis on clinical swallowing evaluation. Participants improved their skills in identifying signs and symptoms of dysphagia, conducting comprehensive evaluations, and interpreting the results. They were introduced to the most widely-used instruments and techniques, the Fiberoptic Endoscopic Evaluation of Swallowing and Videofluoroscopic Swallowing Study, and witnessed real-life demonstrations of the procedures alongside expert interpretation of the results. The attendees were given the opportunity to expand their knowledge on formulating individualized treatment plans, and learned about the International Dysphagia Diet Standardization Initiative, compensation techniques, and evidence-based rehabilitation options, based on the latest research. Interactive case studies allowed participants to apply the newly acquired knowledge, fostering a deeper understanding of practical implementation. Overall, the first day offered an immersive and enriching experience, equipping participants with essential skills and knowledge to address the challenges posed by dysphagia.

Further on, the Course delved into the identification of swallowing disorders, and the crucial role of nutrition in dysphagia with a focus on the specific challenges faced by tracheostomized patients, alongside tracheal-tube management and ventilation, and strategies to safely optimize the swallowing function of affected individuals. A practical session on critical thinking, where participants analyzed complex clinical cases, emphasized the significance of this skill in developing personalized dysphagia management approaches. To enhance practical skills, a hands-on implementation module offered interactive sessions to aid participants in developing approaches tailored to their settings through both the exploration of real-life scenarios under the guidance of the instructor and exercising and refining the skills developed throughout the training. Altogether, the second day of the Dysphagia Course Series became the set up for comprehensive and engaging explorations of dysphagia-related topics, providing attendees with valuable insights and practical tools for their professional settings.

**Figure 2:**
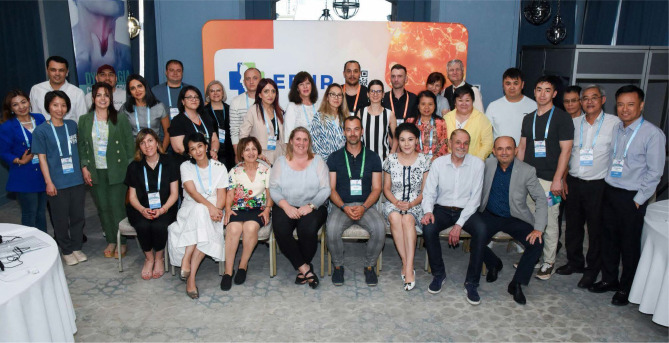
Faculty and participants from the first Dysphagia Course Series, in Tashkent, Uzbekistan

This event presented a resounding success, engaging worldwide specialists and a distinguished faculty (Figure 2) to create a better understanding and improved clinical and collaborative skills for managing dysphagia, a swallowing disorder that affects individuals of all ages and impacts their quality of life. Such specialized training focused on addressing neurological complications through multidisciplinary approaches represents the future in neurorehabilitation education, with the hands-on approaches and the exploration of real-life scenarios bringing added value to the acquired knowledge put into practice in the most dynamic matter.

## DYSPHAGIA COURSE SERIES: THE LYON EXPERIENCE

**Figure 3:**
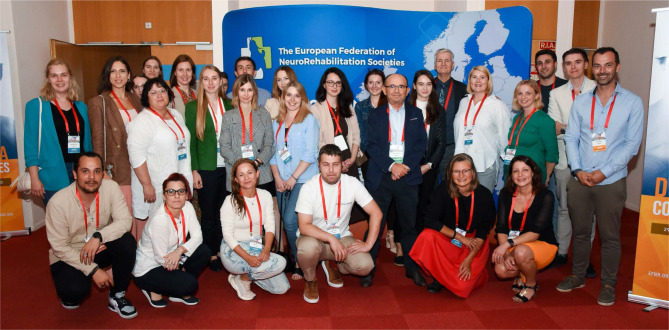
Faculty and participants from the second Dysphagia Course Series, in Lyon, France

The second Dysphagia Course Series (Figure 3) took place in the beautiful city of Lyon, France on the 29^th^ of August 2023. The Course Series welcomes healthcare professionals in speech-language pathology, otolaryngology, neurology, nutritional sciences, and nursing and provides a comprehensive overview and understanding of dysphagia.

In the quest to cultivate a collaborative learning environment, the Dysphagia Course emphasizes the importance of hands-on approaches in a multidisciplinary framework to enhance clinical skills and ultimately improve patient outcomes. The participants were immersed in a dynamic program featuring compelling case studies, expert-led discussions, and interactive activities.

The scientific event delved into the importance of dysphagia, exploring the fundamentals of normal swallowing as the basis for its management. Moreover, the program approached disordered swallowing with a focus on dysphagia following a stroke, the identification of swallowing disorders (screening, clinical swallowing evaluation, instrumental assessment), nutritional aspects, compensation, and rehabilitation. The interactive hands-on session that concluded the event helped participants develop implementation plans for dysphagia management tailored to their respective settings. Facilitated by esteemed experts in the field, Michaela Trapl-Grundschober from the University Clinic Tulln, Austria, and Björn Degen from the Center for Swallowing Disorders Vienna, Austria the one-day intensive course provided attendees with a throughout understanding of dysphagia and its implications.

As dysphagia represents a widely-encountered affection, especially in stroke patients, addressing it heads-on should represent a focus for specialists worldwide. Addressing common neurological affections through a holistic multidisciplinary approach and increasing the quality of life for patients is of utmost importance, as the burden of neurological disorders is on the rise.

